# Patient and nodule characteristics associated with adherence to lung cancer screening in a large integrated healthcare system

**DOI:** 10.1038/s41598-025-15053-1

**Published:** 2025-08-09

**Authors:** Shuang Yang, Muxuan Liang, Hiren J. Mehta, Ramzi G. Salloum, Dejana Braithwaite, Yonghui Wu, Jessica Islam, Xuhong Zhang, Ya-Chen Tina Shih, Jinhai Huo, Jiang Bian, Yi Guo

**Affiliations:** 1 Department of Health Outcomes and Biomedical Informatics, College of Medicine, University of Florida, Gainesville, FL USA; 2 Cancer Informatics Shared Resource, University of Florida Health Cancer Center, Gainesville, FL USA; 3 Department of Biostatistics, College of Public Health and Health Professions, College of Medicine, University of Florida, Gainesville, FL USA; 4 Division of Pulmonary, Critical care, and Sleep Medicine, University of Florida College of Medicine, Gainesville, FL USA; 5 Division of Population Health Sciences, Department of Surgery, College of Medicine, University of Florida, Gainesville, FL USA; 6 University of Florida Health Cancer Center, Gainesville, FL USA; 7 Moffitt Cancer Center, Tampa, FL USA; 8 Department of Computer Science, Luddy School of Informatics, Computing, and Engineering, Indiana University Bloomington, Bloomington, IN USA; 9 Department of Radiation Oncology, School of Medicine, University of California, Los Angeles, Los Angeles, CA USA; 10 Global Real World Evidence, Johnson & Johnson Innovative Medicine, Raritan, NJ USA

**Keywords:** Electronic health records, Real-world evidence, Adherence, Cancer screening, Risk factors

## Abstract

We examined the association of pulmonary nodule characteristics with adherence to follow-up low-dose computed tomography (LDCT) after the initial screening in lung cancer screening. Using 2014–2021 electronic health record data from a large integrated health system, we analyzed adherence to Lung Imaging Reporting and Data System (Lung-RADS) follow-up recommendations, considering socio-demographic, clinical factors, and natural language processing-extracted nodule characteristics. Multivariable logistic regression models assessed the impact of these factors on adherence to follow-up LDCT. Among 2,673 individuals (mean age = 66.8 ± 5.9 years), overall adherence was 27.6%, with rates of 24.2%, 27.5%, 26.7%, and 64.0% for Lung-RADS categories 1–4 A. A race-ethnicity disparity in adherence was observed among category 1, with non-Hispanic blacks less likely to adhere than non-Hispanic whites (OR[95% CI] = 0.59[0.41–0.85]). Among patients in categories 2 to 4 A, category 4 A was significantly more likely to adhere (OR[95% CI] = 3.18[1.86–5.40]) and having more nodules increased adherence (OR[95% CI] = 1.12[1.09–1.14]). Adherence to follow-up LDCT is suboptimal, driven by patient and nodule characteristics, and influenced by how physicians communicated initial CT results. These findings underscore the need for structured screening programs and consistent follow-up protocols to improve adherence and ensure effective lung cancer screening.

## Introduction

Lung cancer is the leading cause of cancer mortality in the United States, accounting for approximately 20% of all cancer-related deaths^1^. The majority (over 70%) of lung cancer cases are diagnosed at advanced stages, significantly reducing the probability of cure and resulting in low survival rates^1^. The National Lung Screening Trial demonstrated that low-dose computed tomography (LDCT) can effectively detect lung cancer early and reduce lung cancer mortality by about 20%^2^. In response, many professional societies and organizations, such as the US Preventive Services Task Force (USPSTF), American Cancer Society, Centers for Medicare and Medicaid Services, National Comprehensive Cancer Network and American Society of Clinical Oncology, have issued guidelines recommending annual lung cancer screening with LDCT for individuals at high-risk for lung cancer^3–8^. For example, the 2013 USPSTF recommends annual LDCT screening for adults aged 55 to 80 years who have a 30 pack-year smoking history and currently smoke or have quit within the past 15 years^3^.

The effectiveness of lung cancer screening is dependent upon adherence to guideline-recommended screening intervals. Adherence in clinical trials such as the National Lung Screening Trial and the Dutch-Belgian Randomized Lung Cancer Screening Trial was high, often surpassing 90%^2,9^. Similarly, the USPSTF assumes perfect adherence to follow-up LDCT when projecting mortality benefits^8^. However, in real-world practice settings, LDCT adherence rates are considerably lower, ranging from 26 to 43%^10–16^. This practice gap may result from variations in institutional practices, diverse populations, and differing definitions of adherence. Identifying factors associated with LDCT adherence is essential for developing effective interventions and guiding policy actions that aim to enhance adherence and the effectiveness of lung cancer screening. Several studies have identified demographic and clinical factors, such as age, race, smoking status, insurance and screening site, that are significantly associated with adherence to initial and subsequent annual LDCT for lung cancer screening^11–17^. Despite these findings, limited studies have developed predictive models of LDCT adherence that incorporate pulmonary nodule findings and characteristics as potential predictors.

The Lung Imaging Reporting and Data System (Lung-RADS^®^) is a quality assurance tool used to categorize lung cancer risk and guide follow-up screening procedures to reduce false-positive findings and standardize lung cancer screening management^18^. Lung-RADS was developed by the American College of Radiology based on lung nodule characteristics detected by LDCT, including nodule size, multiplicity and texture^19^. Although Lung-RADS comprehensively reflects lung cancer risk in a categorical manner, the characteristics of the lung nodules themselves are important additional indicators of malignancy and may influence a patient’s decision to consistently return for follow-up screenings. For example, previous research has reported that nonsolid and part-solid types of nodules from LDCT are more likely to be malignant than solid nodules^20–22^. Additionally, while nodule size is a critical predictor of malignancy, studies have shown that the largest pulmonary nodule in an individual is not always malignant^23^. As the number of nodules increases, the presence of more uncertain characteristics can affect the accuracy of Lung-RADS assessments and influence a patient’s decision to adhere to lung cancer screening recommendations. However, information on pulmonary nodule characteristics is often documented in free-text clinical notes, such as radiology reports in electronic health records (EHR) systems, making it less accessible for research studies.

In the current study, we aimed to build statistical models to examine the demographic, clinical, and pulmonary nodule characteristics associated with follow-up LDCT adherence using both structured and unstructured EHR data from a large integrated health system. We used natural language processing (NLP) tools previously developed and validated to extract pulmonary nodule characteristics from clinical notes for modeling. This model can help identify patients who may be most likely to benefit from interventions aimed at improving lung cancer screening adherence and reducing the burden of lung cancer.

## Results

### Characteristics of study population

**Fig. 1:**
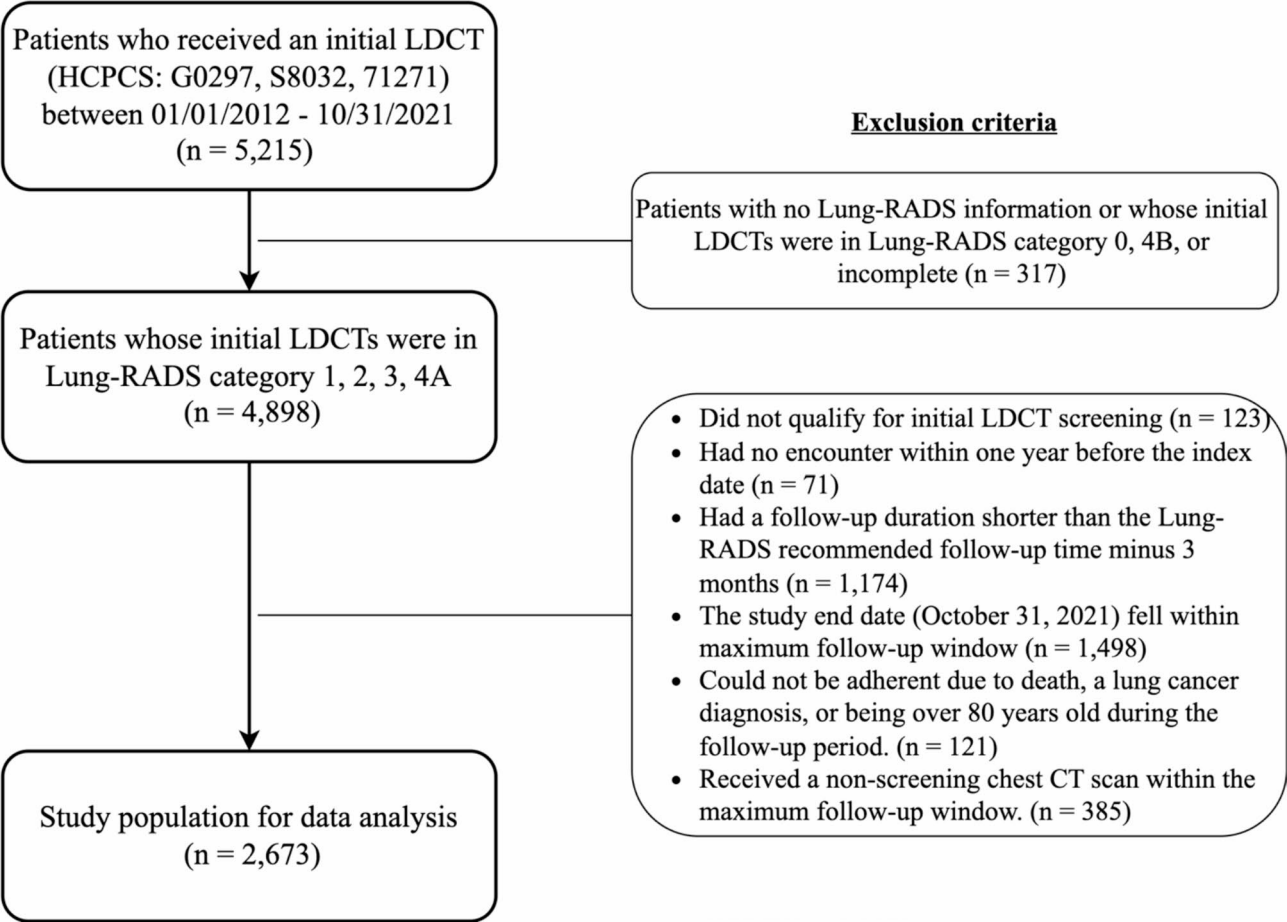
Study population flowchart.

We identified 5,215 patients who received their initial LDCT in the UF IDR data, among whom 4,898 had the initial LDCT in Lung-RADS categories 1 to 4 A. After applying the exclusion criteria, 2,673 individuals (mean age = 66.8 ± 5.9 years) were included in the final data analysis (Fig. 1). We summarized the patient characteristics overall as well as stratified by Lung-RADS category in Table 1. The distribution of the Lung-RADS category in the analytic sample was 47.5%, 42.4%, 5.5%, and 4.7% for categories 1–4 A, respectively. Most of patients were non-Hispanic white (69.3%), residents in urban census tracts (65.8%), and slightly more than half were men (51.4%) or current smokers (54.4%). Over one third of patients had COPD (38.7%) and substantial burden of comorbidities (CCI ≥ 2) (36.9%). About one in four of patients (26.0%) had a family history of cancer. The most common insurance of primary payer was Medicare (64.9%). The median number of nodules identified in the initial LDCT was 3 nodules. The most common values for the nodule characteristics were < 6 mm for nodules size (70.0%), upper for nodule site (39.2%), right lung for nodule laterality (59.8%), and solid for nodule texture (26.7%).

**Table 1:** **Patient characteristics by Lung-RADS category**. Values are n (%) except for number of outpatient visits, number of inpatient visits, number of nodules. ^a^Determined by linking patient’s latest zip-code to the Rural-Urban commuting area (RUCA) codes. ^b^Determined by linking patient’s latest zip-code to the census bureau’s American community survey and categorizing percent population below poverty into 3 groups: < 10%, 10%−19%, or ≥ 20%. ^c^Measured within one year prior to the date of initial lung cancer screening. ^d^Chronic obstructive pulmonary disease ^e^Charlson comorbidity index; 0 = no comorbidity, 1 = some comorbidities, ≥ 2 = a substantial burden of comorbidities. ^f^Other insurance included managed care, worker’s compensation, and other types. ^g^Other site included basilar, lingula, apex, and hilum. ^h^Other laterality included medial, and either left or right, ^i^Other texture included cystic, bubbly, fluids, and mixed.

	Overall (*n* = 2,673)	Category 1 (*n* = 1,269, % = 47.5)	Category 2 (*n* = 1,133, % = 42.4)	Category 3 (*n* = 146, % = 5.5)	Category 4 A (*n* = 125, % = 4.7)	*p*-value
Age (years)						< 0.001
55–59	812 (30.4)	433 (34.1)	313 (27.6)	34 (23.3)	32 (25.6)	
60–64	783 (29.3)	373 (29.4)	327 (28.9)	48 (32.9)	35 (28.0)	
65–69	628 (23.5)	272 (21.4)	284 (25.1)	48 (32.9)	24 (19.2)	
70–80	450 (16.8)	191 (15.0)	209 (18.4)	16 (11.0)	34 (27.2)	
Sex						0.558
Women	1,300 (48.6)	612 (48.2)	563 (49.7)	71 (48.6)	54 (43.2)	
Men	1,373 (51.4)	657 (51.8)	570 (50.3)	75 (51.4)	71 (56.8)	
Race-ethnicity						0.208
Non-Hispanic white	1,852 (69.3)	847 (66.7)	808 (71.3)	107 (73.3)	90 (72.0)	
Non-Hispanic black	706 (26.4)	359 (28.3)	284 (25.1)	33 (22.6)	30 (24.0)	
Other	115 (4.3)	63 (4.9)	41 (3.6)	6 (4.1)	5 (4.0)	
Smoking status						0.122
Former	1,220 (45.6)	603 (47.3)	509 (44.8)	55 (37.7)	56 (44.8)	
Current	1,453 (54.4)	667 (52.6)	626 (55.2)	91 (62.3)	69 (55.2)	
Marital status						0.051
Married	1,158 (43.3)	540 (42.6)	500 (44.1)	69 (47.3)	49 (39.2)	
Single	570 (21.3)	283 (22.3)	217 (19.1)	31 (21.2)	39 (31.2)	
Divorced	945 (35.4)	446 (35.1)	416 (36.7)	46 (31.5)	37 (29.6)	
Family cancer history						
Yes	694 (26.0)	327 (25.8)	301 (26.6)	30 (20.6)	36 (28.8)	0.393
Census tract rurality^a^						0.086
Non-Urban	914 (34.2)	416 (32.8)	393 (34.6)	50 (34.3)	55 (44.0)	
Urban	1,759 (65.8)	853 (67.2)	740 (65.3)	96 (65.7)	70 (56.0)	
Census tract poverty^b^						0.113
< 10%	5221 (19.5)	256 (20.2)	225 (19.9)	27 (18.5)	13 (10.3)	
10% − 19%	1,327 (49.7)	610 (48.1)	564 (49.8)	77 (52.7)	76 (60.8)	
≥ 20%	688 (25.7)	345 (27.2)	277 (24.5)	35 (24.0)	31 (24.8)	
Unknown	137 (5.1)	58 (4.6)	67 (5.9)	7 (4.8)	5 (4.0)	
Number of outpatients visits^c^ (Median (IQR))	9.0 (4–16)	9.0 (4–17)	9.0 (4–16)	8.5 (4–14)	9.0 (4–16)	0.489
Number of inpatient visits^c^ (Median (IQR))	0.7 (0–1)	0.5 (0–1)	0.6 (0–1)	0.6 (0–1)	1.1 (0–2)	0.456
COPD^c, d^	1,033 (38.7)	489 (38.5)	419 (37.0)	63 (43.2)	62 (49.6)	0.031
CCI^e^						0.561
0	865 (32.4)	429 (33.8)	355 (31.3)	47 (32.2)	34 (27.2)	
1	821 (30.7)	376 (29.6)	351 (31.0)	48 (32.9)	46 (36.8)	
≥ 2	987 (36.9)	464 (36.6)	427 (37.7)	51 (34.9)	45 (36.0)	
Primary Payer for initial LDCT						0.077
Medicare	1,734 (64.9)	810 (63.8)	731 (64.5)	104 (71.2)	89 (71.2)	
Commercial	417 (15.6)	205 (16.2)	174 (15.3)	26 (17.8)	12 (9.6)	
Medicaid and all other insurances^f^	522 (19.5)	254 (20.0)	228 (20.1)	16 (11.0)	24 (19.2)	
Nodule Characteristics						
Number of nodules (Median (IQR))	3.0 (1–7)	-	5.0 (2–9)	6.0 (3–12)	10.0 (5–18)	< 0.001
Size						< 0.001
< 6 mm	962 (70.0)	-	915 (80.7)	37 (25.4)	10 (8.0)	
6–8 mm	184 (13.4)	-	94 (8.3)	74 (50.7)	16 (12.8)	
> 8 mm	229 (16.6)	-	99 (8.7)	31 (21.2)	99 (79.2)	
Site						0.098
Lower	468 (33.3)	-	353 (31.1)	71 (48.6)	44 (35.2)	
Middle	128 (9.1)	-	109 (9.6)	10 (6.8)	9 (7.2)	
Upper	550 (39.2)	-	464 (40.9)	44 (30.1)	42 (33.6)	
Other^g^	258 (18.4)	-	207 (18.3)	21 (14.4)	30 (24.0)	
Laterality						0.012
Left	509 (36.3)	-	416 (36.7)	54 (37.0)	39 (31.2)	
Right	840 (59.8)	-	678 (59.8)	81 (58.5)	81 (67.3)	
Bilateral	35 (2.5)	-	27 (2.4)	5 (3.4)	3 (2.4)	
Other^h^	20 (1.4)	-	12 (1.1)	6 (3.0)	2 (1.8)	
Texture						< 0.001
Calcified	351 (25.3)	-	321 (28.3)	13 (8.9)	19 (15.2)	
Ground glass	161 (11.9)	-	141 (12.4)	9 (6.2)	14 (11.2)	
Noncalcified	171 (12.3)	-	141 (12.4)	21 (14.4)	10 (8.0)	
Soft	171 (12.1)	-	124 (10.9)	21 (14.4)	26 (20.8)	
Solid	371 (26.7)	-	271 (23.9)	60 (41.1)	39 (31.2)	
Other^i^	164 (11.6)	-	125 (11.0)	22 (15.1)	17 (13.6)	
Adherent to Lung-RADS guideline						
Yes	737 (27.6)	307 (24.2)	311 (27.4)	39 (26.7)	80 (64.0)	< 0.001

We observed significant differences in certain patient characteristics across the Lung-RADS categories. A higher percentage of category 1 patients were in the youngest age group (55–59 years), whereas a higher percentage of category 4 patients were in the oldest age group (70–80 years; overall *p* for age < 0.001). Additionally, a higher percentage of patients in categories 3 and 4 A had COPD compared to those in categories 1 and 2 A (overall *p* for COPD = 0.031).

Regarding the primary outcome, the overall rate of adherence to Lung-RADS recommended follow-up LDCT was 27.6%. This rate differed significantly by Lung-RADS category (*p* < 0.001), with the lowest rate observed in category 1 patients (24.2%), and the highest in category 4 A patients (64.0%).

### Results from multivariable regression models

We summarized results from the multivariable logistic models in Table 2. In the model for patients in Lung-RADS category 1, non-Hispanic blacks were significantly less likely to be adherent to follow-up LDCT compared to non-Hispanic whites (OR = 0.59, 95% CI = 0.41–0.85). Having a higher number of outpatient visits in the year before the initial LDCT was associated with greater adherence to follow-up LDCT (OR = 1.01, 95% CI = 1.00-1.03). Additionally, patients whose initial LDCT was covered by Medicaid or other insurance types had lower adherence to follow-up LDCT compared with those who had their initial LDCT covered by Medicare (OR = 0.65, 95% CI = 0.43–0.99).

**Table 2:** **Odds ratios from logistic models for predicting adherence to follow-up LDCT**.^a^Determined by linking patient’s latest zip-code to the Rural-Urban commuting area (RUCA) codes. ^b^Determined by linking patient’s latest zip-code to the census bureau’s American community survey and categorizing percent population below poverty into 3 groups: < 10%, 10%−19%, or ≥ 20%. ^c^ Measured within one year prior to the date of initial lung cancer screening. ^d^Charlson comorbidity index; 0 = no comorbidity, 1 = some comorbidities, ≥ 2 = a substantial burden of comorbidities. ^e^Other insurance included commercial insurance, managed care, worker’s compensation, and other types. ^f^Other site included basilar, lingula, apex, and hilum. ^g^Other laterality included medial, and either left or right, ^h^Other texture included cystic, bubbly, fluids, and mixed. OR = odds ratio; CI = confidence interval; COPD = chronic obstructive pulmonary disease.

Variables	Category 1, Adjusted OR (95% CI)	*p*-value	Category 2–4 A, Adjusted OR (95% CI)	*p*-value
Lung-RADS				
3 vs. 2	-	-	0.80 (0.48–1.33)	0.382
4 A vs. 2	-	-	3.18 (1.86–5.40)	< 0.001
Age (years)				
60–64 vs. 55–59	1.29 (0.90–1.84)	0.165	0.80 (0.56–1.14)	0.221
65–69 vs. 55–59	1.23 (0.81–1.88)	0.324	0.90 (0.60–1.35)	0.693
70–80 vs. 55–59	1.14 (0.72–1.82)	0.573	1.04 (0.67–1.61)	0.861
Sex				
Men vs. Women	1.02 (0.77–1.34)	0.908	1.12 (0.86–1.47)	0.393
Race				
Non-Hispanic black vs. Non-Hispanic white	0.59 (0.41–0.85)	0.005	0.94 (0.66–1.33)	0.746
Other vs. Non-Hispanic white	1.01 (0.56–1.83)	0.972	0.98 (0.48–1.95)	0.897
Smoking status				
Current vs. Former	1.05 (0.80–1.37)	0.744	0.91 (0.70–1.18)	0.482
Marital status				
Single vs. Married	0.74 (0.51–1.09)	0.129	0.82 (0.57–1.18)	0.281
Divorced vs. Married	1.05 (0.77–1.44)	0.765	0.82 (0.65–1.20)	0.195
Family cancer history				
Yes or No	1.23 (0.90–1.67)	0.198	0.88 (0.65–1.20)	0.444
Census tract rurality^a^				
Urban vs. Non-Urban	1.33 (0.97–1.82)	0.074	1.04 (0.76–1.41)	0.801
Census tract poverty^b^				
10%−19% vs. < 10%	0.68 (0.34–1.35)	0.271	1.77 (0.89–3.49)	0102
≥ 20% vs. < 10%	0.76 (0.40–1.44)	0.394	1.72 (0.91–3.24)	0.091
Unknown vs. < 10%	0.55 (0.27–1.10)	0.088	1.30 (0.65–2.61)	0.455
Number of Outpatient visits^c^	1.01 (1.00-1.03)	0.027	1.01 (1.00-1.03)	0.032
Number of Inpatient visits^c^	0.74 (0.54–1.02)	0.065	0.98 (0.76–1.27)	0.899
COPD^c^	1.27 (0.91–1.79)	0.158	1.14 (0.82–1.58)	0.442
CCI^d^				
1 vs. 0	0.96 (0.65–1.40)	0.821	0.78 (0.55–1.13)	0.191
≥ 2 vs. 0	0.87 (0.57–1.31)	0.491	0.69 (0.46–1.03)	0.071
Primary Payer for initial LDCT				
Commercial vs. Medicare	0.73 (0.47–1.13)	0.156	0.68 (0.44–1.05)	0.081
Medicaid and other insurance^e^ vs. Medicare	0.65 (0.43–0.99)	0.042	0.86 (0.58–1.26)	0.420
Nodule Characteristics				
Number of nodules	-	-	1.12 (1.09–1.14)	< 0.001
Size				
6–8 mm vs. <6 mm	-	-	0.66 (0.42–1.02)	0.063
> 8 mm vs. <6 mm	-	-	0.97 (0.63–1.48)	0.889
Site				
Middle vs. Lower	-	-	1.23 (0.87–1.74)	0.249
Upper vs. Lower	-	-	1.06 (0.69–1.46)	0.810
Other^f^ vs. Lower	-	-	1.22 (0.69–2.12)	0.491
Texture				
Ground glass vs. Calcified	-	-	1.11 (0.71–1.74)	0.649
Noncalcified vs. Calcified	-	-	0.81 (0.51–1.28)	0.367
Soft vs. Calcified	-	-	0.81 (0.50–1.30)	0.361
Solid vs. Calcified	-	-	0.92 (0.64–1.32)	0.661
Other^g^ vs. Calcified	-	-	0.97 (0.68–1.39)	0.767
Laterality				
Left vs. bilateral	-	-	0.61 (0.28–1.35)	0.221
Right vs. bilateral	-	-	0.63 (0.30–1.40)	0.264
Other^h^ vs. bilateral	-	-	0.64 (0.15–2.72)	0.536

In the model for patients in Lung-RADS categories 2–4 A, patients in category 4 A were significantly more likely to adhere to follow-up LDCT compared to those in category 2 (OR = 3.18, 95% CI = 1.86–5.40). Having a higher number of outpatient visits in the year before the initial LDCT was associated with greater adherence to follow-up LDCT (OR = 1.01, 95% CI = 1.00-1.03). Regarding nodule characteristics, a higher number of nodules was associated with greater adherence to follow-up LDCT (OR = 1.12, 95% CI = 1.09–1.14). We also tested interactions between nodule characteristics and Lung-RADS categories 2–4 A and found no significant effect modification, indicating that these associations were consistent across categories.

## Discussion

In this study, we extracted EHR data from a large integrated healthcare system and examined the demographic, clinical, and nodule characteristics associated with patients’ adherence to follow-up LDCT. The pulmonary nodule characteristics included number of nodules, nodule size, texture, laterality, and site, which were extracted from radiology reports using previously validated NLP tools. The rate of adherence to follow-up LDCT was 27.6% overall and 24.2%, 27.4%, 26.7%, and 64.0% for patients in Lung-RADS categories 1–4 A, respectively. We observed racial/ethnic disparity in adherence to follow-up LDCT among category 1 patients, with non-Hispanic blacks less likely to be adherent than non-Hispanic whites. Among patients in categories 2 to 4 A, category 4 A patients were significantly more likely to be adherent and having a higher number of nodules was associated with greater adherence.

Our findings highlight that adherence rates increased across Lung-RADS categories, with category 4 A patients significantly more likely to adhere to follow-up LDCT compared to those in category 2. This aligns with the expectation that patients with more suspicious findings perceive a higher risk and are more likely to follow recommendations. However, adherence remains suboptimal in lower-risk groups, possibly due to false reassurance after a negative initial result or inconsistent physician communication. Standardized communication protocols within structured screening programs are essential to ensure patients receive clear, consistent messaging, particularly for those with indeterminate findings, and to reinforce the importance of continued follow-up.

Previous studies have reported that patients’ adherence to follow-up LDCT ranges from 26 to 43% ^10–16^. The observed overall adherence rate in the current study of 27.6% is comparable to these published rates, all of which are significantly lower than rates reported in clinical trials and screening programs for other cancers, such as breast and colorectal cancer^24,25^. This suggests that effective interventions are needed to improve adherence to follow-up LDCT and thus the effectiveness of lung cancer screening. It has been suggested that interventions such as frequent follow-up reminders, navigator support, and educational materials that emphasize the benefits of lung cancer screening through the screening program can be considered to enhance adherence to follow-up LDCT^15,16,26^. In the current study, we also found that demographic factors such as race-ethnicity, insurance of primary payer, and regular prior healthcare utilization were associated with adherence to follow-up LDCT, which is consistent with previous findings^17,26^. These findings indicate that patient subgroups at higher risk of being non-adherent must be identified to efficiently deploy intervention and resources for improving the effectiveness of lung cancer screening. Additionally, increasing Medicaid coverage of LDCT for lung cancer screening may be an effective way to improve access and adherence to lung cancer screening.

Few studies have examined the impact of pulmonary nodule characteristics on patients’ adherence to lung cancer screening guidelines. We found only one such study in which the authors reported the distribution of the nodules’ characteristics based on manually reviewed and extracted nodule characteristics from 260 patients^12^. They found a median nodule number of one, with a median size of 3 mm, predominantly solid nodules located in the upper and right lobes, which is comparable to the nodule distributions in our study population. However, nodule characteristics were not considered in prior prediction models of adherence, possibly due to the small sample size. Information on nodule characteristics is usually documented in radiology reports as text. Research studies using this information often rely on manual review and extraction of nodule characteristics by radiologists for data analysis, which is time-consuming and limits the study sample size^12,27,28^. We used NLP technology to efficiently extract nodule characteristics from unstructured data and were able to include a considerably sized study population. Our findings suggest that the number of nodules is important factors to consider when designing interventions for improving lung cancer screening adherence.

Despite the strengths of using both structured and unstructured EHR data from a large integrated healthcare system, our study has a few limitations. First, our study population came from a healthcare system in Florida, findings from our study may not be generalizable to patients from other geographic locations. Second, we used a ± 3-month window around the recommended follow-up time interval to determine adherence of follow-up LDCT, which may not capture all follow-up LDCTs performed. This approach could have favored an underestimation of follow-up LDCT adherence. Third, while the NLP tools we developed for extracting nodule characteristics from unstructured data have excellent performance and many advantages, misclassification errors cannot be eliminated. Fourth, due to data limitations, we did not have access to complete information on pack-year history or time since quit smoking, so we identified the study population based on age and smoking status alone, which may have affected adherence rates by including patients with lower perceived risk or different follow-up behaviors compared to strictly guideline-eligible individuals. Finally, adherence may have been underestimated since patients could have undergone LDCTs outside the UF Health system.

## Conclusions

Adherence to follow-up LDCT for lung cancer screening is suboptimal and is influenced by certain patient demographic and pulmonary nodule characteristics. Our results support the value of integrating variables extracted from unstructured data using advanced NLP technology for more comprehensive data analysis. Our findings underscore the need for targeted interventions and suggest potential strategies for designing interventions to improve adherence to lung cancer screening.

## Methods

### Data source and study population

We obtained 2012–2021 patient-level EHR data from the University of Florida (UF) Health Integrated Data Repository (IDR), a clinical data warehouse aggregating patient information from UF’s various clinical and administrative systems, including the Epic EHR system. The IDR contains more than one billion observational data elements from more than two million patients, encompassing structured data such as patient demographics, diagnoses, medical procedures, vital signs, laboratory tests, and medications, as well as unstructured clinical narratives such as discharge summaries, order notes, and pathology reports. This study was approved by the UF Institutional Review Board (IRB). All methods were performed in accordance with relevant guidelines and regulations.

The UF Health lung cancer screening program was implemented in 2014, shortly after the USPSTF recommendations for LDCT screening were established. The program adheres to national guidelines, which are updated in accordance with USPSTF revisions. Additionally, the Lung-RADS classification system, introduced by the American College of Radiology in 2014, was adopted early by the UF Health lung cancer screening program and has been used consistently to guide follow-up recommendations. Patients were typically referred for lung cancer screening by their primary care providers or pulmonary physicians, who assess eligibility based on guideline criteria. LDCT results were communicated to patients through the electronic medical record system, where complete radiology reports were accessible. However, there was no standardized institutional protocol for communicating results. As such, communication practices varied by providers—ranging from brief summaries of the Lung-RADS category and recommended follow-up to detailed discussions of specific nodule findings. This variability may have influence patients’ understanding of their risk and their adherence to follow-up recommendations.

We identified patients who underwent at least one LDCT procedure between October 1, 2014 and October 31, 2021 in UF Health IDR data using Current Procedural Terminology (CPT) codes based on their effective date range (S8032, effective from October 1, 2014-September 30, 2016; G0297, effective from February 5, 2015-December 31, 2020; and 71271, effective from January 1, 2021 onwards). For each patient, the date of the first LDCT was defined as the index date. We excluded patients: (1) who did not qualify for LDCT screening (i.e., were not current or former smokers, or whose age at the initial LDCT did not meet the USPSTF eligibility criteria—age 55–80 per the 2013 guideline if before March 2021, and age 50–80 per the 2021 guideline if on or after March 2021); (2) who had no encounter records within one year before the index date, to ensure sufficient prior data for measuring baseline characteristics; (3) whose follow-up period (from the index date to their last EHR visit) was shorter than the Lung-RADS recommended follow-up time minus 3 months; (4) whose follow-up period (from the index date to the study end date, October 31, 2021) was shorter than the Lung-RADS recommended follow-up time plus 3 months; (5) who could not be adherent due to death, a lung cancer diagnosis, or being order than 80 years old during the follow-up period; (6) who had received a non-screening chest CT scan within the maximum follow-up window, as these scans could preclude adherence to Lung-RADS-defined follow-up LDCT protocols and lead to misclassification of adherence status.

Due to data limitations, pack-year history and time since quitting smoking were unavailable, therefore, eligibility for LDCT screening was determined based on age and smoking status alone.

### Study outcome

The primary outcome was whether a patient who had received an initial LDCT was adherent to Lung-RADS recommended follow-up schedule for LDCT. Specifically, the Lung-RADS recommended follow-up interval is 12 months for categories 1 (i.e., negative) and 2 (i.e., benign appearance or behavior), 6 months for category 3 (i.e., probably benign), and 3 months for category 4 A (i.e., suspicious). For Lung-RADS categories 4B and 4X (i.e., highly suspicious), immediate chest CT or PET/CT with or without biopsy is recommended, but no standard follow-up is prescribed^29^. We included patients whose initial LDCT was in Lung-RADS categories 1, 2, 3, and 4 A which involve standard follow-up rather than immediate interventions. Lung-RADS categories for the initial LDCT were extracted from lung cancer screening order narratives using our previously developed rule-based approach^30^. Lung-RADS categories are often documented in radiology reports with specific patterns, including numbers and letters (e.g., “Lung-RADS category: 4A”). Our rule-based approach, using regular expressions to capture these patterns, achieved an F1-score of 0.998. Being adherent to follow-up LDCT was defined as undergoing the second LDCT within ± 3 months of the recommended follow-up time interval after the initial LDCT.

### Predictors of interest

The predictors of interest included socio-demographic, clinical and pulmonary nodule characteristics. The socio-demographic characteristics included age at index date, sex, race-ethnicity, census tract-level rurality and poverty, smoking status, insurance of primary payer for the initial LDCT, baseline healthcare utilization, and marital status, whereas the clinical characteristics included family cancer history, baseline chronic pulmonary disease (COPD) status, and Charlson comorbidity index (CCI)^31^. Census tract-level rurality was determined by linking patient’s latest zip-code in the EHRs to the Rural-Urban Commuting Area (RUCA) codes^32^ and categorizing patients as urban (RUCA code 1) or non-urban (RUCA code 2–10) residents. Census tract-level poverty, defined as the percentage of the population below poverty line, was determined by linking patients’ latest zip-codes to the Census Bureau’s American Community Survey and categorizing them into 3 groups: < 10%, 10%−19%, ≥ 20%. Smoking status (i.e., current or former smoker) and marital status (i.e., married/partnered, single, or other) were determined using the most recent EHR status before the index date. Insurance of primary payer for the initial LDCT was categorized as Medicare, commercial, Medicaid or other (e.g., charity, worker’s compensation, managed care, federal/state/local government insurance, self-pay). Baseline healthcare utilization was measured using the numbers of outpatient and inpatient visits within one year prior to the index date. Family history of all cancer (ICD-9: V16; ICD-10: Z80) was extracted from structured EHR data prior to the index date. Additionally, baseline COPD (ICD-9: 490–496; ICD-10: J40-J44) and CCI were extracted from EHR data within 12 months prior to the index date. We calculated the CCI following the modified algorithm by Klabunde et al.^31^. CCI was categorizing into 3 groups: no comorbidity (CCI = 0), some comorbidities (CCI = 1), a substantial burden of comorbidities (CCI ≥ 2).

Pulmonary nodule characteristics included Lung-RADS categories (extracted using rule-based algorithms mentioned previously) and nodule characteristics, both extracted using NLP from unstructured EHR data. Five categories of nodule characteristics were extracted from clinical notes and included in this study as predictor of adherence to follow-up LDCT: the number of the nodules, the largest nodule size (0, < 6 mm, 6–8 mm, > 8 mm), nodule texture (calcified, ground glass, noncalcified, soft, solid, other), laterality (left, right, bilateral, other), site (lower, middle, upper, other). The pulmonary nodules and associated nodule characteristics were extracted from radiology reports using NLP system with state-of-the-art transformer models, which we developed and validated previously using UF Health EHRs^30^. The NLP system integrated the robustly optimized BERT approach (RoBERTa)-mimic model for concept extraction, A Lite BERT (ALBERT)-base model for the relation identification, and the RoBERTa-mimic model for negation detection. Our end-to-end NLP system for extracting pulmonary nodule and nodule characteristics achieved an excellent F1-score of 0.8869 (precision = 0.8345 and recall = 0.9464).

#### Statistical analysis

We calculated summary statistics to describe the study characteristics in the overall population and by Lung-RADS category. Continuous variables were presented as means with standard deviations for those following a normal distribution or as medians with interquartile ranges (25th and 75th percentiles) for those that were skewed. Categorical variables were summarized using frequencies and percentages. Normality of continuous data was assessed using the Kolmogorov-Smirnov test. Differences in study characteristics across Lung-RADS categories were evaluated using analysis of variance (ANOVA) or the Kruskal-Wallis test for continuous variables, and the Chi-squared or Fisher’s exact test for categorical variables. For variables with missing values, we created an “unknown” category and included it in both univariate comparisons and in the regression models to retain the full analytic sample. Other variables had no missing values. We built univariable and multivariable logistics regression models to examine the factors associated adherence to screening. Separate models were built for patients in Lung-RADS category 1 and those in categories 2–4 A because over 90% of the patients in category 1 had no nodules. Pulmonary nodule characteristics were used as predictors in the model for patients in Lung-RADS categories 2–4 A only. To assess whether the associations between nodule characteristics and adherence differed by Lung-RADS category (2–4 A), we tested interaction terms between each nodule characteristic and Lung-RADS category. All effects were estimated as odds ratios (ORs) with 95% confidence intervals (CIs). Two-sided p-values were calculated for all statistics, considering a significance level of 0.05. Data processing and management were conducted using python 3.9.4. Statistical analyses were conducted using SAS 9.4 (SAS Institute Inc., Cary, NC, USA).

## Data Availability

The EHR dataset curated from UF IDR cannot be released due to HIPAA regulations and require IRB approval for access. Analysis codes are available upon request from the corresponding author.

## References

[1] Siegel, R. L., Miller, K. D., Wagle, N. S. & Jemal, A. Cancer statistics, 2023. *CA Cancer J. Clin.* **73**, 17–48 (2023). 36633525 10.3322/caac.21763

[2] National Lung Screening Trial Research Team. The National lung screening trial: overview and study design. *Radiology* **258**, 243–253 (2011). 21045183 10.1148/radiol.10091808 PMC3009383

[3] Moyer, V. A. Preventive services task force. Screening for lung cancer: U.S. Preventive services task force.recommendation statement. *Ann. Intern. Med.* **160**, 330–338 (2014). 24378917 10.7326/M13-2771

[4] US Preventive Services Task Force. Screening for lung cancer: US preventive services task force recommendation statement. *JAMA* **325**, 962–970 (2021). 33687470 10.1001/jama.2021.1117

[5] Wender, R. et al. American cancer society lung cancer screening guidelines. *CA Cancer J. Clin.* **63**, 107–117 (2013). 23315954 10.3322/caac.21172 PMC3632634

[6] Services, C. M. Decision Memo for Screening for Lung Cancer with Low Dose Computed Tomography (LDCT)(CAG-00439 N). in *Centers for Medicare and Medicaid Services*.

[7] Wood, D. E. et al. NCCN guidelines^®^ insights: lung cancer screening, version 1.2022. *J. Natl. Compr. Canc Netw.* **20**, 754–764 (2022). 35830884 10.6004/jnccn.2022.0036

[8] Begnaud, A., Hall, T. & Allen, T. Lung cancer screening with low-dose CT: implementation amid changing public policy at one health care system. *Am. Soc. Clin. Oncol. Educ. Book.* **36**, e468–e475 (2016). 10.1200/EDBK_159195 27249755

[9] de Koning, H. J. et al. Reduced lung-cancer mortality with volume CT screening in a randomized trial. *N Engl. J. Med.* **382**, 503–513 (2020). 31995683 10.1056/NEJMoa1911793

[10] Hirsch, E. A., New, M. L., Brown, S. P., Barón, A. E. & Malkoski, S. P. Patient reminders and longitudinal adherence to lung cancer screening in an academic setting. *Ann. Am. Thorac. Soc.* **16**, 1329–1332 (2019). 31339348 10.1513/AnnalsATS.201902-152RL

[11] Bastani, M. et al. Factors associated with lung cancer screening adherence among patients with negative baseline CT results in a community health care setting. *J. Am. Coll. Radiol.* **19**, 232–239 (2022). 34861204 10.1016/j.jacr.2021.10.010

[12] Barbosa, E. J. M. Jr, Yang, R. & Hershman, M. Real-world lung cancer CT screening performance, smoking behavior, and adherence to recommendations: Lung-RADS category and smoking status predict adherence. *AJR Am. J. Roentgenol.* **216**, 919–926 (2021). 32755178 10.2214/AJR.20.23637

[13] Sakoda, L. C. et al. Patterns and factors associated with adherence to lung cancer screening in diverse practice settings. *JAMA Netw. Open.* **4**, e218559 (2021). 33929519 10.1001/jamanetworkopen.2021.8559 PMC8087957

[14] Hirsch, E. A. et al. Determinants associated with longitudinal adherence to annual lung cancer screening: A retrospective analysis of claims data. *J. Am. Coll. Radiol.* **18**, 1084–1094 (2021). 33798496 10.1016/j.jacr.2021.03.003 PMC8349785

[15] Wernli, K. J. et al. Understanding patient and clinical stakeholder perspectives to improve adherence to lung cancer screening. *Perm J* **25**, (2021). 10.7812/TPP/20.295 PMC8817936 35348073

[16] Lin, Y. et al. Factors associated with nonadherence to lung cancer screening across multiple screening time points. *JAMA Netw. Open.* **6**, e2315250 (2023). 37227725 10.1001/jamanetworkopen.2023.15250 PMC10214032

[17] Lopez-Olivo, M. A. et al. Patient adherence to screening for lung cancer in the US: A systematic review and meta-analysis. *JAMA Netw. Open.* **3**, e2025102 (2020). 33196807 10.1001/jamanetworkopen.2020.25102 PMC7670313

[18] Chelala, L. et al. Lung-RADS version 1.1: challenges and a look ahead, from the AJR special series on radiology reporting and data systems. *AJR Am. J. Roentgenol.* **216**, 1411–1422 (2021). 33470834 10.2214/AJR.20.24807

[19] Jonas, D. E. et al. Screening for lung cancer with low-dose computed tomography: updated evidence report and systematic review for the US preventive services task force. *JAMA* **325**, 971–987 (2021). 33687468 10.1001/jama.2021.0377

[20] Dyer, S. C., Bartholmai, B. J. & Koo, C. W. Implications of the updated lung CT screening reporting and data system (Lung-RADS version 1.1) for lung cancer screening. *J. Thorac. Dis.* **12**, 6966–6977 (2020). 33282402 10.21037/jtd-2019-cptn-02 PMC7711402

[21] Tamura, M., Shimizu, Y., Yamamoto, T., Yoshikawa, J. & Hashizume, Y. Predictive value of one-dimensional mean computed tomography value of ground-glass opacity on high-resolution images for the possibility of future change. *J. Thorac. Oncol.* **9**, 469–472 (2014). 24736068 10.1097/JTO.0000000000000117

[22] Kitazawa, S. et al. Three-dimensional mean CT Attenuation value of pure and part-solid ground-glass lung nodules May predict invasiveness in early adenocarcinoma. *Clin. Radiol.* **74**, 944–949 (2019). 31630766 10.1016/j.crad.2019.09.130

[23] McWilliams, A. et al. Probability of cancer in pulmonary nodules detected on first screening CT. *N Engl. J. Med.* **369**, 910–919 (2013). 24004118 10.1056/NEJMoa1214726 PMC3951177

[24] Ferreira, C. S., Rodrigues, J., Moreira, S., Ribeiro, F. & Longatto-Filho, A. Breast cancer screening adherence rates and barriers of implementation in ethnic, cultural and religious minorities: A systematic review. *Mol. Clin. Oncol.* **15**, 139 (2021). 34055354 10.3892/mco.2021.2301 PMC8145341

[25] Fisher, D. A. et al. Utilization of a colorectal cancer screening test among individuals with average risk. *JAMA Netw. Open.* **4**, e2122269 (2021). 34473259 10.1001/jamanetworkopen.2021.22269 PMC8414191

[26] Bastani, M. et al. A predictive model for lung cancer screening nonadherence in a community setting health-care network. *JNCI Cancer Spectr* **7**, (2023). 10.1093/jncics/pkad019 PMC10097452 37027213

[27] Moseson, E. M. et al. Patient and clinician characteristics associated with adherence. A cohort study of veterans with incidental pulmonary nodules. *Ann. Am. Thorac. Soc.* **13**, 651–659 (2016). 27144794 10.1513/AnnalsATS.201511-745OC

[28] Mortani Barbosa, E. J. Jr & Kelly, K. Statistical modeling can determine what factors are predictive of appropriate follow-up in patients presenting with incidental pulmonary nodules on CT. *Eur. J. Radiol.* **128**, 109062 (2020). 32422551 10.1016/j.ejrad.2020.109062

[29] Martin, M. D., Kanne, J. P., Broderick, L. S., Kazerooni, E. A. & Meyer, C. A. Lung-RADS: pushing the limits. *Radiographics* **37**, 1975–1993 (2017). 29053407 10.1148/rg.2017170051

[30] Yang, S. et al. Extracting pulmonary nodules and nodule characteristics from radiology reports of lung cancer screening patients using transformer models. *J. Healthc. Inf. Res.*10.1007/s41666-024-00166-5 (2024). 10.1007/s41666-024-00166-5 PMC11310180 39131104

[31] Klabunde, C. N., Warren, J. L. & Legler, J. M. Assessing comorbidity using claims data: an overview. *Med. Care*. **40**, IV–26 (2002). 12187165 10.1097/00005650-200208001-00004

[32] Cromartie, J. & Documentation https://www.ers.usda.gov/data-products/rural-urban-commuting-area-codes/documentation/

